# FGL2 as a predictive biomarker for prognosis and immunotherapy in bladder cancer

**DOI:** 10.7150/ijms.91874

**Published:** 2024-05-27

**Authors:** Zhengnan Huang, Zeyi Wang, Chengdang Xu, Yilin Yan, Xiangqian Cao, Fang Zhang, Bing Shen

**Affiliations:** 1 Department of Urology, Tongji Hospital, School of Medicine, Tongji University, Shanghai 200065, China.; 2 Department of Urology, Huadong Hospital, Fudan University, Shanghai, China. 200040, China.; 3 Department of Urology, Shanghai General Hospital, Shanghai Jiaotong University School of Medicine, Shanghai 200080, China.; 4 Shanghai Tenth People's Hospital, School of Medicine, Tongji University, Shanghai 200072, China.

**Keywords:** bladder cancer, FGL2, prognosis, immunotherapy, tumor microenvironment.

## Abstract

**Background:** Metastasis and immunosuppression result in unfavorable prognosis in bladder cancer (BLCA). FGL1 and FGL2 are two members of the fibrinogen-related proteins family, but their potential effects on BLCA remain elusive.

**Methods:** The expression profile of FGL1 and FGL2 in BLCA was analyzed in multiple databases. Furthermore, the expression of FGL2 was validated in BLCA tissues. The predictive capability of FGL2 was evaluated by Kaplan-Meier analysis, univariate analysis, and multivariate Cox regression. A nomogram model was constructed based on FGL2 expression and clinicopathological parameters for clinical practice. Gene Ontology (GO), Kyoto Encyclopedia of Genes and Genomes (KEGG), and Gene Set Enrichment Analyses (GSEA) were performed to investigate enrichment in the biological processes. In addition, the correlation between FGL2 and immunological characteristics in the BLCA tumor microenvironment (TME), including tumor-infiltrating immune cells (TICs), cancer-immunity cycles, immune checkpoint molecules (ICPs), immunophenoscores (IPS), and response to anti-PD-L1 immunotherapy was further analyzed.

**Results:** FGL2 was found to be downregulated in BLCA due to hypermethylation of the FGL2 promoter region, which was associated with an unfavorable prognosis. Moreover, BLCA patients with high FGL2 expression exhibited better response to immunotherapy.

**Conclusions:** Our research revealed that FGL2 was downregulated in BLCA and was negatively correlated with DNA methylation. High FGL2 expression was confirmed as an independent risk for prognosis. Moreover, FGL2 is a promising indicator for the response to immunotherapy in patients with BLCA.

## Introduction

Bladder cancer (BLCA) is the 11th most common human malignancy in the world, with almost 550, 000 new cases diagnosed annually 1, 2. Despite the variety of available treatment modalities, such as surgery, chemotherapy, radiotherapy, and immunotherapy, the overall treatment effects remain unsatisfactory due to tumor recurrence, metastasis, and resistance to chemotherapy drugs, resulting in a low five-year survival rate 3-5. Hence, dependable predictive biomarkers are required for precise prognosis and for developing new molecular targets for accurate treatment of BLCA.

Growing evidence demonstrated that fibrinogen-like protein-1 (FGL1) and fibrinogen-like protein-2 (FGL2), two members of the fibrinogen-related proteins (FREP) family, play a crucial role in modulating the function of immune cells and in the development of multiple cancers 6. FGL1, also known as hepatocyte-derived fibrinogen-like protein-1 (HFREP1) 7, is a hepatocellular secreted protein initially cloned from human hepatocellular carcinoma 8. Under physiological conditions, FGL1 is specifically produced by liver parenchymal cells and regulates liver metabolism. Following hepatocyte poisoning or surgery, FGL1 is significantly upregulated and acts as a mitotic active factor to promote hepatocyte proliferation 9-11. FGL2, also named FGL2 prothrombinase, is cloned from cytotoxic T lymphocytes and shares 36% homology with fibrinogen β and γ chains 12, 13. FGL2 not only exerts prothrombinase activity directly catalyzing the conversion of prothrombin into thrombin 14, but also performs potent immunoregulatory functions in a variety of diseases, including autoimmune disorders, xenograft rejection, viral-induced inflammation, chronic obstructive pulmonary disease, and tumor growth 15-19.

Interestingly, FGL1 may exert either anti-tumor activities or tumor-promoting effects, depending on the tumor type. For example, the overexpression of FGL1 in gastric cancer was negatively correlated with prognosis, promoting cell proliferation and migration 20. Meanwhile, FGL1 depletion was found to accelerate the development of hepatocellular carcinoma through an Akt-dependent mechanism, supporting the role of FGL1 as a tumor suppressor 21. Similar to FGL1, FGL2 plays opposite roles in different tumor types. For instance, FGL2 was found to act as an immune regulator, promoting glioblastoma progression through immunosuppression mechanisms 22, 23. In contrast, FGL2 exerted anti-tumor effects and served as a protective beneficial biomarker in lung adenocarcinoma 24. However, the diagnostic value and potential function of FGL1 and FGL2 in BLCA remain incompletely understood.

This study employed multifaceted bioinformatics analysis methods to examine the expression profile and prognostic value of FGL1 and FGL2 in BLCA. Ultimately, FGL2 was identified as a promising predictive indicator for BLCA immunotherapy.

## Materials and Methods

### Expression of FGL1 and FGL2 in BLCA

The expression difference of FGL1 and FGL2 between cancer tissues and their corresponding normal tissues was analyzed in Oncomine database (https://www.oncomine.org/resource/login.html). TCGA-BLCA (https://www.tcga-data.nci.nih.gov/tcga) and GEO (http://www.ncbi.nlm.nih.gov/geo; GSE13507 and GSE19915) were further applied to confirm the expression difference of FGL2 in BLCA. UALCAN (http://ualcan.path.uab.edu/) was utilized to quest the correlation of gene expression with clinical parameters.

### Tissue specimens

Paraffin-embedded tissues of patients with BLCA and corresponding clinical data, fresh BLCA tissues and adjacent non-tumor tissues were collected from the Department of Urology, Shanghai General Hospital. All patients included in this study have signed a prior informed consent, and the study was approved by the ethical committee of the Shanghai General Hospital.

### Cell lines and cell culture

Human BLCA cell lines (T24, J82, UM-UC-3, and TCC-SUP) were acquired from the Chinese Academy of Sciences Shanghai cell bank in China. T24 cells were cultivated in RPMI-1640 (Gibco), while J82, UM-UC-3, and TCC-SUP cells were cultivated in Eagle's Minimum Essential Medium (Gibco). All culture media were supplemented with 10% fetal bovine serum (FBS) and penicillin/streptomycin. The cells were kept in an environment saturated with 5% CO_2_ at 37 °C. In addition, the cells were exposed to a concentration of 5 μM 5-Aza-CdR (Sigma-Aldrich, USA) for the designated duration. Subsequently, the cells were harvested for RNA and protein isolation.

### RNA isolation and qRT-PCR

Cellular total RNA extraction was carried out utilizing TRIzol reagent (TaKaRa), followed by reverse transcription into complementary DNA (cDNA) using the HiScript III RT SuperMix Kit (vazyme). Quantitative PCR reactions were conducted employing the ChamQ SYBR qPCR Master Mix (vazyme). The primers (5' to 3') used in this study were as follows, β-actin-F: CATGTACGTTGCTATCCAGGC; β-actin-R: CTCCTTAATGTCACGCACGAT; FGL2-F: CAGGCTGATGACAACGGAGAC; FGL2-R: TCCAGGCGACCATGAAGTACA.

### Western blotting

Western blotting was conducted as previously described 25. Briefly, RIPA buffer with phosphatase and protease inhibitors was used to extract the total protein in tissues. Proteins were separated by SDS-PAGE and transferred onto a PVDF membrane. After blocking in 5% non-fat milk with PBST, primary antibodies against FGL2 (1:500, 11827-1-AP, Proteintech) and GAPDH (1:1000, #5174, Cell Signaling Technology) were applied. Finally, the NcmECL Ultra kit (NCM biotech, Shanghai, China) was used to visualize the western blots.

### Immunohistochemistry (IHC)

IHC staining was conducted following the standard methods as previously reported 26. Briefly, paraffin sections were deparaffinized in xylene for antigen retrieval, followed by the incubation with anti-FGL2 (1:50, 11827-1-AP, Proteintech) antibody at 4°C overnight. The DAB visualization kit (MaixinBio, Fuzhou, China) was then applied to visualize the localization of the antigen and counterstain sections with hematoxylin.

### Methylation analysis

To appraise the correlation between FGL2 expression and methylation status in BLCA, the methylation level of FGL2 and its correlation with clinicopathological characteristics were analyzed in UALCAN (http://ualcan.path.uab.edu/). The Spearman method was applied to measure the strength of the correlation between FGL2 mRNA levels and its related methylation sites.

### Prognostic analysis

The overall survival was appraised by Kaplan-Meier survival analysis using the “survival” package and “survminer” package of R software. Univariate and multivariate Cox analysis was also performed by means of the “Survival” package.

### Construction and validation of nomogram

FGL2 expression and clinicopathological parameters were utilized to establish the nomogram by using the R package “rms” and “survival”. Calibration curves were drawn to estimate the authenticity of the nomogram.

### Functional enrichment analysis

FGL2 low- and high-expression groups were categorized based on the RNA-seq data of TCGA-BLCA, with the median value as the cut-off. The R package “limma” was used to identify differentially expressed genes (DEGs) with the screening criteria |log2FC| > 1 and P < 0.05. GO, KEGG, and GSEA analyses were conducted for enrichment analysis, which were performed using the R package “clusterprofiler”.

### Immunity Analysis

CIBERSORT algorithm was utilized to examine the proportion of 22 immune cells in BLCA samples of different FGL2 expression subgroups. The cancer-immune cycle reflects the anticancer immune response and consists of seven steps: release of cancer cell antigens (Step 1), cancer antigen presentation (Step 2), priming and activation (Step 3), trafficking of immune cells to tumors (Step 4), infiltration of immune cells into tumors (Step 5), recognition of cancer cells by T cells (Step 6) and killing of cancer cells (Step 7) 27. The activities of these steps determine the fate of the tumor cells. Xu *et al.* evaluated the activities of these steps using a single sample gene set enrichment analysis (ssGSEA) based on the gene expression of individual samples 28. The correlation of FGL2 with the enrichment scores of immunotherapy-predicted pathways was further investigated. Gene Set Variation Analysis (GSVA) was then carried out to calculate the enrichment scores of each sample. Immunophenoscores (IPS) of BLCA patients was retrieved from the Cancer Immunome Atlas (TCIA, https://tcia.at/home), the association of FGL2 expression with IPS was investigated by the Wilcoxon rank-sum test. IMvigor210 cohort, an immune-related cohort for the treatment of BLCA, was obtained from http://researchpub.Gene.com/imvigor210corebiologies/ 29, which was utilized to further investigate the potential capability of FGL2 in forecasting the clinical response to immunotherapy.

## Results

### Transcriptional level of FGL1 and FGL2 in BLCA patients

**Figure 1:**
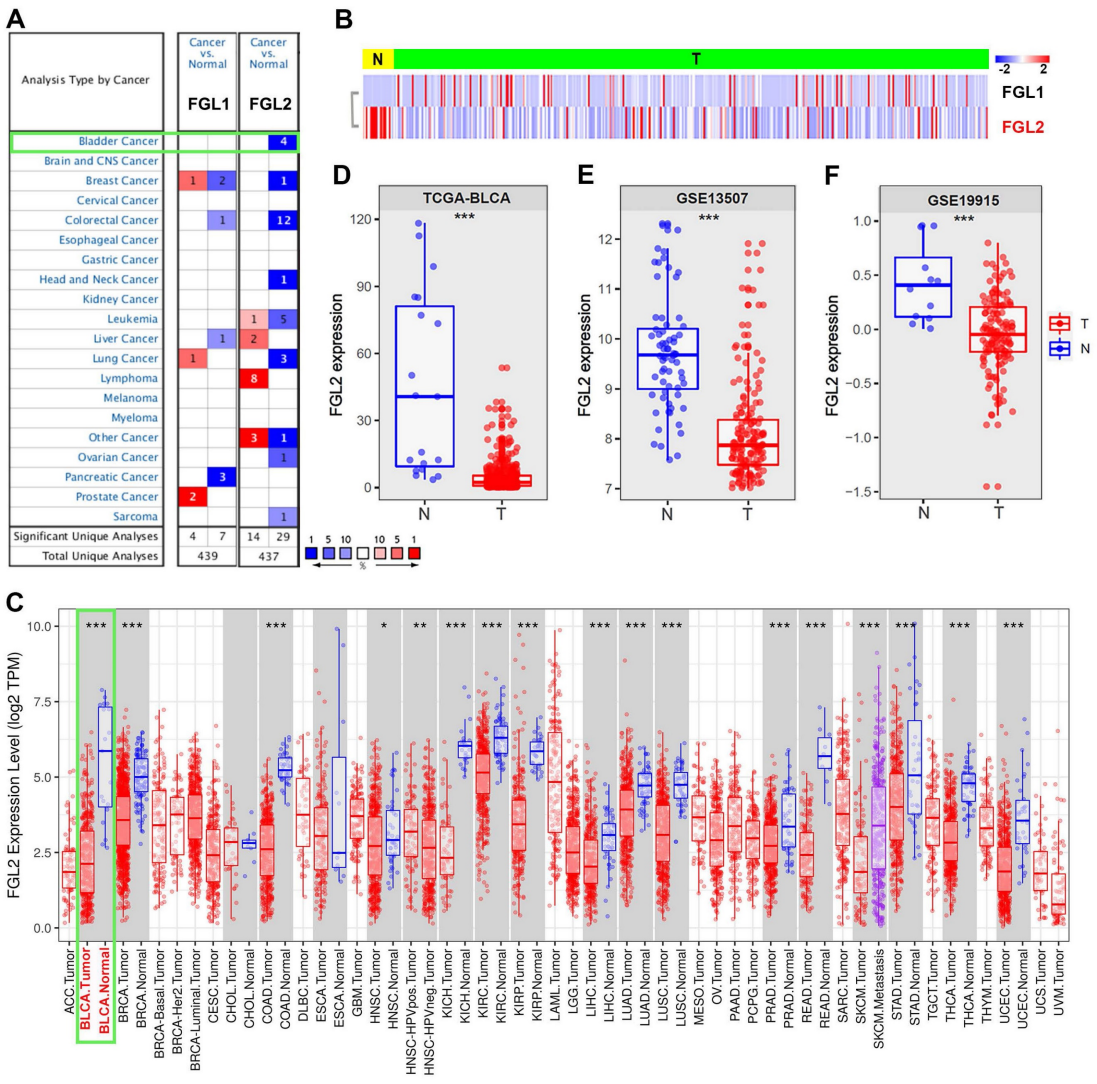
** The expression of FGL2 in pan-carcinoma and BLCA. (A)** Transcriptional expression of FGL1 and FGL2 in 20 different types of cancer and normal samples in Oncomine database.** (B)** Heatmap of the transcriptional level of FGL1 and FGL2 between normal and BLCA tissues in TCGA.** (C)** Expression of FGL2 in pan-carcinoma and their corresponding normal controls in TIMER database. (**D-F**) Boxplots of FGL2 expression difference between BLCA tissues (T) and nontumorous counterparts (N) in TCGA-BLCA, GSE13507, and GSE19915. *p < 0.05, **p < 0.01, ***p < 0.001.

The expression of FGL1 and FGL2 was analyzed in the Oncomine database to explore the potential value of FGL1 and FGL2 in BLCA patients. As shown in Figure 1A, the expression of FGL1 and FGL2 in 20 types of cancers and their normal counterparts were measured. The mRNA expression of FGL2 was significantly lower in BLCA, while FGL1 expression showed no significant difference compared to their normal counterparts. A heatmap was used to display the transcriptional levels of FGL1 and FGL2 between normal and BLCA tissues in TCGA (Figure 1B). The results from the TIMER database also showed the expression of FGL2 in pan-carcinoma, indicating downregulated FGL2 expression in bladder tumor tissues (Figure 1C). Boxplots of FGL2 expression further confirmed decreased mRNA levels of FGL2 in BLCA based on the data from TCGA-BLCA, GSE13507, and GSE19915 (Figure 1D-F). Furthermore, samples from the UALCAN database were analyzed based on sample type, molecular subtype, lymph node metastasis, and cancer stage, revealing a significantly lower FGL2 expression in BLCA compared to normal controls (Figure S1A-D). However, extremely low FGL1 expression was observed in BLCA, showing no significant difference in expression between normal and tumor tissues (Figure S2A-E). Collectively, these results indicated abnormal FGL2 expression in BLCA, whereas the FGL1 expression levels were unremarkable.

### FGL2 protein was significantly downregulated in BLCA

**Figure 2:**
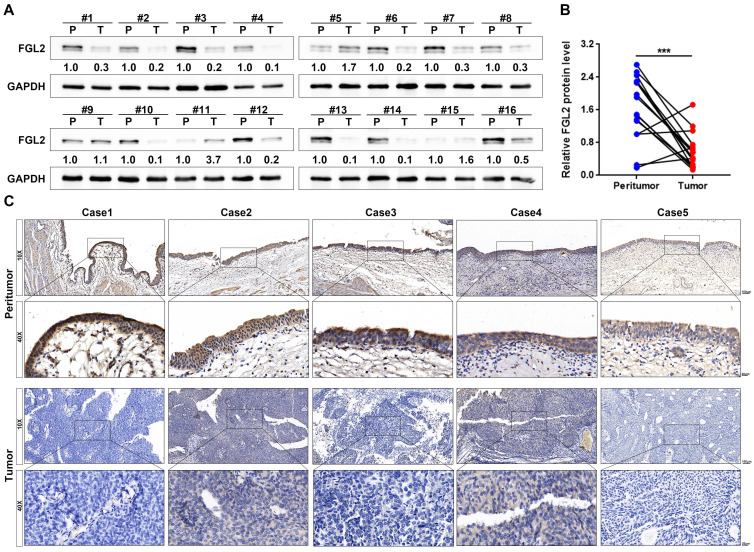
** The protein expression of FGL2 in BLCA patients. (A, B)** FGL2 protein level in 16 paired tumoral tissues (T) and peritumoral tissues (P). **(C)** Representative IHC images of FGL2 expression in peritumoral tissues and tumoral tissues. Scale bar, 100 and 20 μm. ***p < 0.001.

To further assess the aberrant FGL2 expression in BLCA, western blotting and IHC were performed to detect the FGL2 protein levels. The western blotting results revealed that FGL2 expression was significantly lower in BLCA tissues than in peritumoral tissues (Figure 2A and B). Subsequently, a total of 71 BLCA tissues were subjected to IHC for further analysis. The representative images of IHC staining display the FGL2 protein expression in peritumoral and tumoral tissues, as shown in Figure 2C. FGL2 was mainly localized in the cytoplasm of peritumoral cells, while no or weak FGL2 staining was observed in tumoral cells. Collectively, these findings further demonstrated the downregulation of FGL2 in BLCA patients.

### Correlation of FGL2 expression with prognosis in BLCA

**Figure 3:**
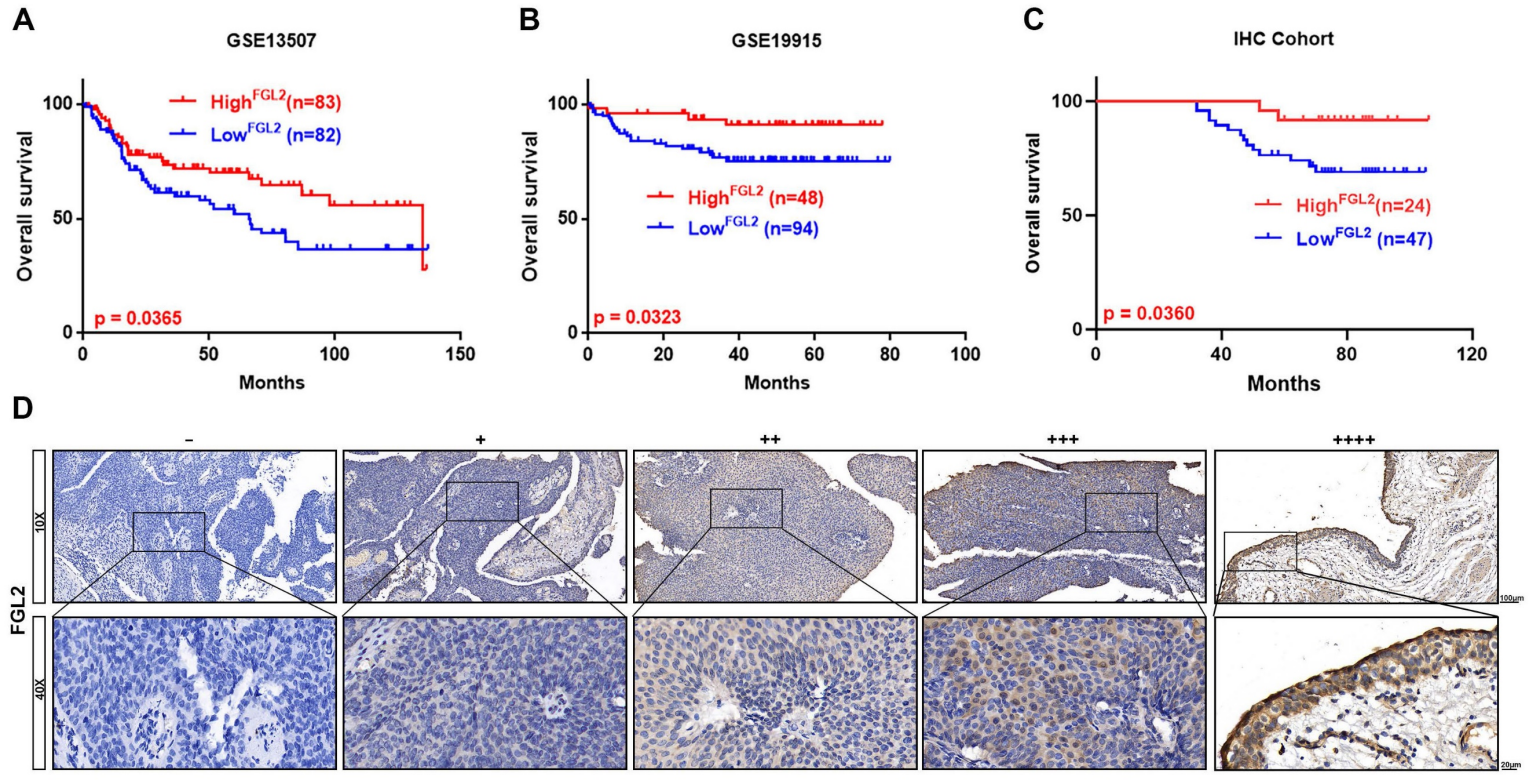
** Low FGL2 expression portended poor prognosis in BLCA patients. (A-C)** Kaplan-Meier curve analysis of overall survival of BLCA patients in GES13507, GSE19915 and IHC cohort. **(D)** Representative IHC images of different FGL2 staining intensities in BLCA tissues. Scale bar, 100 and 20 μm.

Next, Kaplan-Meier survival analysis was conducted to examine the potential prognostic value of FGL1 and FGL2 in BLCA. Analysis of the GSE13507 and GSE19915 cohorts revealed that decreased expression of FGL2 was negatively associated with overall survival (p < 0.05; Figure 3A and B), whereas FGL1 expression showed no significant correlation with clinical prognosis (Figure S2F). Consistently, the IHC results also validated that lower FGL2 protein levels were associated with poor prognosis (p < 0.05; Figure 3C). The representative images of IHC staining of different FGL2 staining intensities are displayed in Figure 3D.

**Figure 4:**
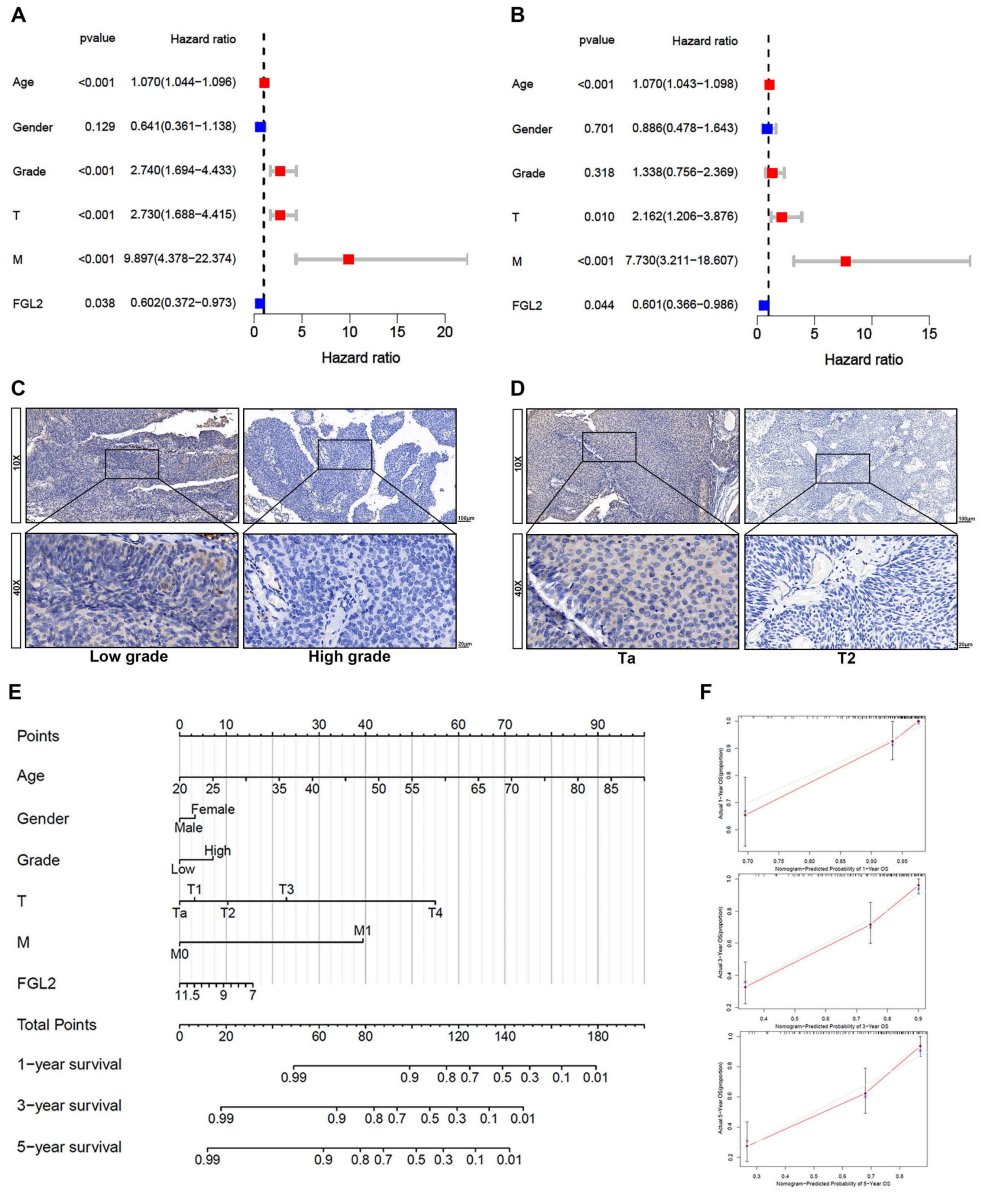
** The prognostic value of FGL2 in BLCA patients. (A, B)** Univariate (A) and multivariate (B) Cox regression analyses of the FGL2 expression with clinical features. **(C, D)** Representative IHC images of the FGL2 protein expression in indifferent pathological grade and stage.** (E)** Nomogram for predicting the overall survival of BLCA patients.** (F)** The calibration plots of 1-year, 3-year, and 5 year- survival of BLCA patients.

The results of the univariate and multivariate Cox analyses of the GSE13507 dataset indicated that FGL2 could be applied as an independent prognostic indicator in BLCA (Figure 4A and B). Further analysis revealed that the FGL2 protein levels were strongly associated with the T stage and tumor grade (Table 1). The representative images of FGL2 protein expression from the IHC analysis revealed notable distinctions across different pathological grades and stages (Figure 4C and D). Thereafter, a prognostic nomogram was constructed to offer a quantitative approach to predicting the prognosis of BLCA patients. The nomogram provides a convenient method to evaluate the survival probability of individual patients (Figure 4E). The model was validated by ideal calibration curves and exhibited great prediction accuracy (Figure 4F). In summary, these findings indicated that FGL2 could act as an independent prognostic biomarker for BLCA.

Collectively, the above results revealed that FGL2, but not FGL1, was unusually silenced in BLCA; downregulation of FGL2 was associated with an unfavorable prognosis, indicating that FGL2 might play an essential role in the progression of BLCA.

### Increased FGL2 methylation levels in BLCA

**Figure 5:**
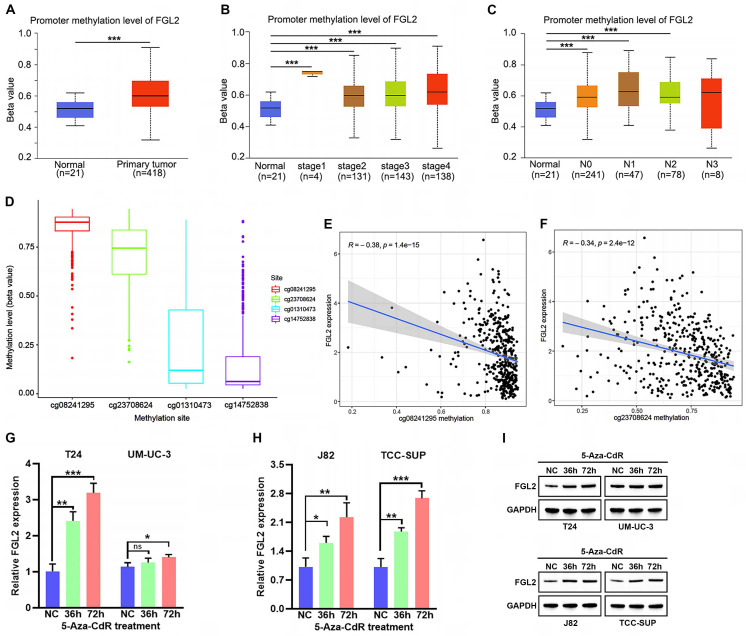
** Analysis of FGL2 methylation status in BLCA. (A-C)** Boxplots of FGL2 promoter methylation profile based on sample types **(A)**, tumor stage **(B)** and node metastasis status **(C)** in UALCAN database. **(D)** The beta values of FGL2 CpG sites in 450 k. **(E, F)** The correlation between the methylation level of FGL2 CpG sites and FGL2 mRNA expression.** (G-I)** FGL2 mRNA and protein expression in BLCA cell lines after treated with 5-Aza-CdR for the indicated times. ns. not significant, *p < 0.05, **p < 0.01, ***P<0.001.

Hypermethylation of CpG sites in promoters frequently leads to transcriptional silencing of genes 30, 31. Considering the frequent downregulation of FGL2 in BLCA, FGL2 expression was speculated to be repressed by promoter methylation. To confirm this hypothesis, the methylation levels of FGL2 in the UALCAN database. As shown in Figure 5A, the total methylation value of FGL2 in the BLCA tissues was significantly higher relative to non-tumorous tissues. Moreover, the methylation level of FGL2 was significantly increased in BLCA patients with advanced tumor stage and lymph node metastasis (Figure 5B and C). Subsequently, the methylation status of FGL2 was further explored by analyzing the methylation value of the CpG sites. The beta values of FGL2 CpG sites in 450 k were displayed in Figure 5D. Among them, cg08241295 and cg23708624 had the higher methylation value. Additionally, the methylation levels of cg08241295 and cg23708624 were negatively associated with FGL2 mRNA expression (Figure 5E and F). To further explore the potential role of promoter methylation in regulating FGL2 transcription in BLCA, BLCA cell lines were treated with the DNA methylation inhibitor agent 5-Aza-CdR. The findings revealed elevated levels of FGL2 mRNA and protein expression following 5-Aza-CdR treatment (Figure 5G-I). Collectively, the above results demonstrated that DNA hypermethylation, a major epigenetic modification, potentially inhibited FGL2 expression at the transcriptional level.

### Function enrichment analysis of FGL2-related genes in BLCA

**Figure 6:**
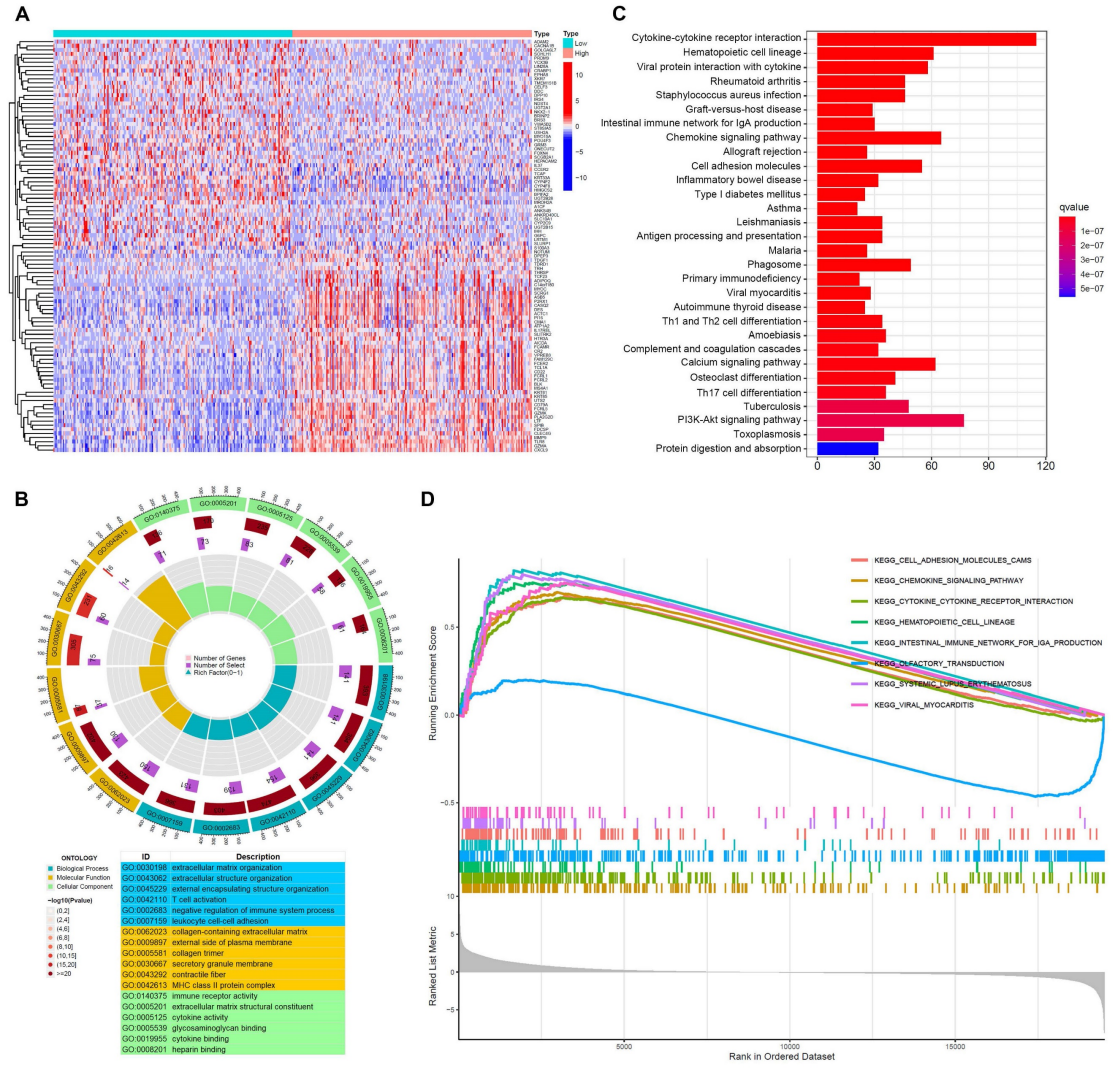
**Function enrichment analysis of FGL2 related genes in BLCA. (A)** Heatmap of the top 100 DEGs between FGL2 high and low subgroups.** (B)** GO enrichment analysis of DEGs (BP: biological progress; CC: cellular component; MF: molecular function). **(C)** KEGG enrichment analysis of DEGs. **(D)** GSEA results showing the top eight significant pathways.

To investigate the underlying role of FGL2 in BLCA, an enrichment analysis of the DEGs was performed based on FGL2 expression. The heatmap displayed the top 100 DEGs of FGL2 (Figure 6A). GO functional analysis revealed that the DEGs of FGL2 expression were primarily associated with T cell activation, negative regulation of the immune system process, collagen-containing extracellular matrix, and immune receptor activity (Figure 6B). Furthermore, KEGG analysis indicated that the DEGs of FGL2 were principally involved in immune-related pathways, such as cytokine-cytokine receptor interaction, intestinal immune network for IgA production, and antigen processing and presentation (Figure 6C). Consistently, GSEA results also showed that cytokine-cytokine receptor interaction and intestinal immune network for IgA production were remarkably enriched (Figure 6D). Collectively, the above results highlighted that FGL2 might be involved in the immune microenvironment in BLCA.

### FGL2 is involved in tumor immunity in BLCA

**Figure 7:**
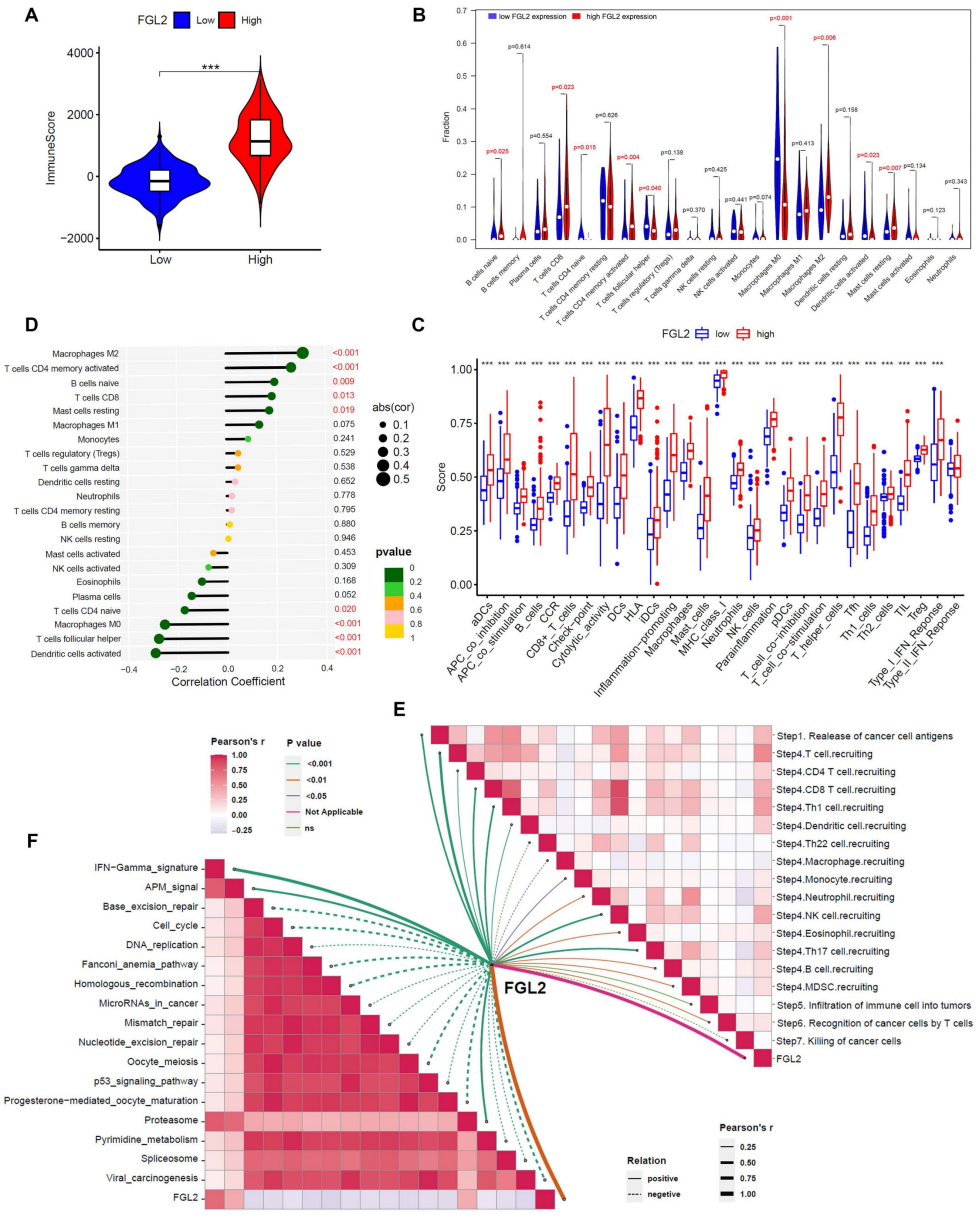
** FGL2 is involved in tumor immunity in BLCA. (A)** The correlation of FGL2 expression with ImmuneScore.** (B)** Difference in the proportions of immune cell type in BLCA with low or high FGL2 expression.** (C)** The association of FGL2 expression with immune cell types and immune-related functions.** (D)** The correlation of FGL2 expression with TICs. **(E)** The correlation of FGL2 with the steps of the cancer-immunity cycle. **(F)** The correlation of FGL2 with the enrichment scores of immunotherapy-predicted pathways. ***P<0.001.

Considering the close association between FGL2 and immune-related biological activities, the potential function of FGL2 in regulating the tumor immune microenvironment was explored. First, the relationship between FGL2 expression and ImmuneScore was analyzed, indicating that patients with high FGL2 expression exhibited higher ImmuneScore (Figure 7A). Second, the CIBERSORT algorithm was utilized to analyze the difference in the proportions of TICs in high- and low-FGL2 expression groups. The results revealed significant differences in naive B cells, CD8+ T cells, CD4+ naive T cells, activated CD4+ memory T cells, follicular helper T cells, macrophage M0, macrophage M2, activated dendritic cells, and resting mast cells between the high and low FGL2 expression groups (Figure 7B). FGL2 expression was significantly associated with most immune-related functions or immune cell types (Figure 7C). Thirdly, the correlation between FGL2 and 22 kinds of TICs was explored, revealing that macrophage M2, activated CD4+ memory T cells, naive B cells, CD8+ T cells, and resting mast cells had a positive correlation with FGL2. In contrast, activated dendritic cells, follicular helper T cells, macrophage M0, and CD4+ naive T cells showed a strong negative correlation (Figure 7D). The activities of the cancer-immunity cycle comprehensively reflect the functions of the immune regulatory system. In this study, FGL2 was found to be positively correlated with the critical steps of the cancer-immunity cycle, including the release of cancer cell antigens (Step 1) and trafficking of immune cells to tumors (Step 4) (CD8 T cell recruiting, Th1 cell recruiting, DC recruiting, NK cell recruiting, and TH17 recruiting) (Figure 7E). Subsequently, the correlation between FGL2 and the predicted immune checkpoint blockade response-related signatures was analyzed. The results suggested that FGL2 was positively correlated with the enrichment scores for immunotherapy-related positive signatures, such as the IFN γ signature (Figure 7F).

### Correlation of FGL2 with the clinical response to immunotherapy in BLCA

**Figure 8:**
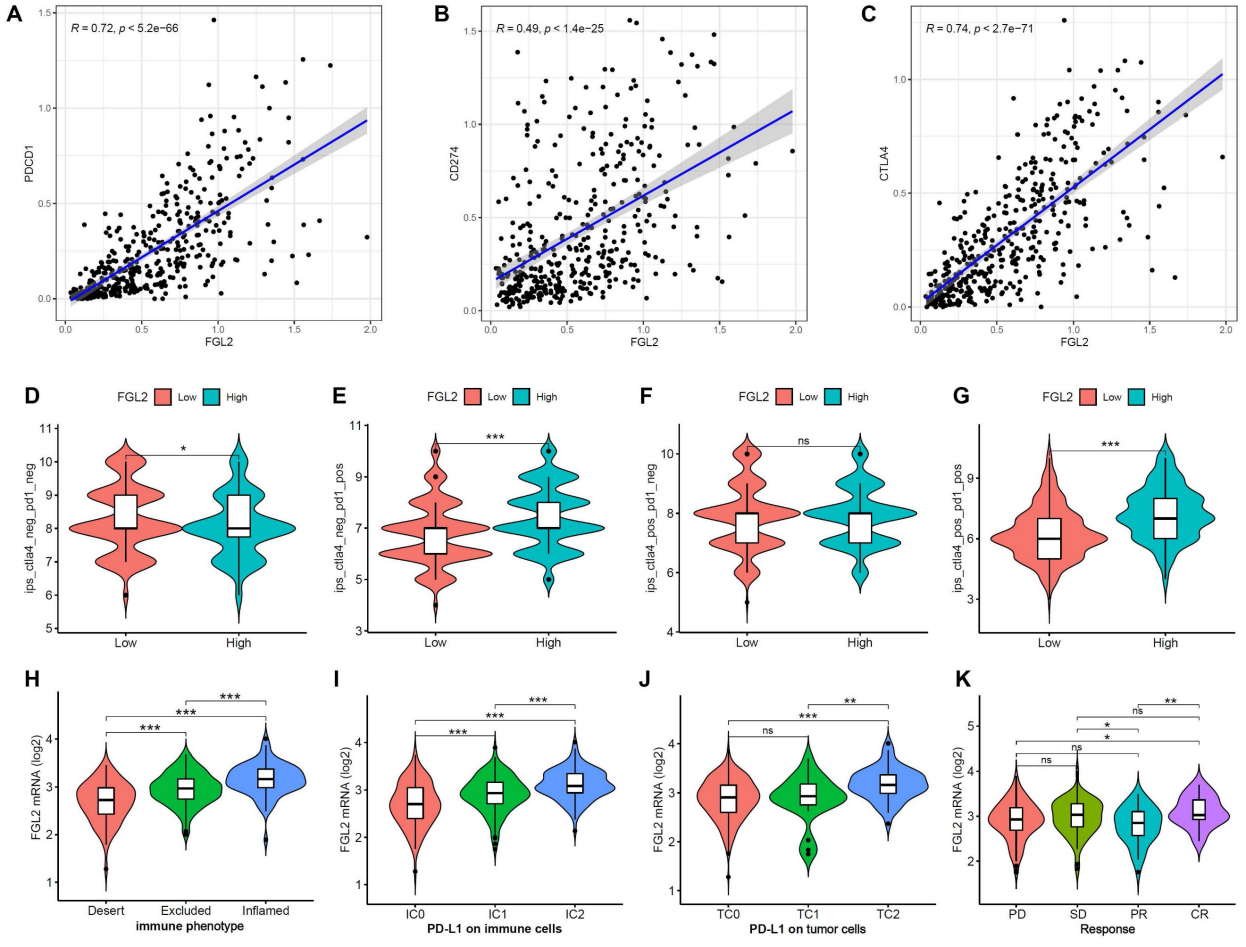
**The role of FGL2 in anti-PD-L1 immunotherapy. (A-C)** The correlation of FGL2 expression of with PDCD1 (PD-1), CD274 (PDL-1) and CTLA4 in TCGA-BLCA.** (D-G)** The correlation of FGL2 expression with Immunophenoscores (IPS) in BLCA patients. **(H)** Expression differences of FGL2 among tumor immune phenotypes in the IMvigor210 cohort. **(I, J)** Expression differences of PD-L1 on immune cells and tumor cells between high- and low-FGL2 groups in the IMvigor210 cohort. **(K)** The correlation of FGL2 with the clinical response of cancer immunotherapy in the IMvigor210 cohort. SD, stable disease; PD, progressive disease; CR, complete response; PR, partial response. ns. not significant, *p < 0.05, **p < 0.01, ***p < 0.001.

To evaluate the prognostic value of FGL2 for immunotherapy response, the correlation between FGL2 expression and ICPs was examined. The results indicated that FGL2 expression exhibited a significantly positive correlation with ICPs (Figure S3), including PDCD1 (PD-1), CD274 (PDL-1), and CTLA4 (Figure 8A-C). Moreover, analysis of the correlation between FGL2 expression and IPS revealed that patients with high FGL2 expression exhibited higher IPS on anti-PD1 and anti-CTLA4 therapy (Figure 8D-G). A previous study reported that higher IPS was positively correlated with enhanced immunogenicity 32, suggesting that FGL2 could be a favorable signature for predicting the immunotherapy effect in BLCA patients. Next, the IMvigor210 cohort, an immune-related cohort for the treatment of BLCA, was analyzed to further investigate the potential capability of FGL2 in forecasting the clinical response to immunotherapy. In the IMvigor210 cohort, lower FGL2 expression was observed in the desert phenotype (patients with this phenotype were less likely to benefit from immunotherapy), IC0 (immune cells with the lowest PD-L1 values), and TC0 (tumor cells with the lowest PD-L1 values) groups (Figure 8H-J). These results suggested that patients with low FGL2 expression were less likely to benefit from immunotherapy. Furthermore, the therapeutic response to anti-PD-L1 immunotherapy was explored, and the results showed that patients with high FGL2 expression exhibited a remarkably enhanced response to anti-PD-L1 immunotherapy (Figure 8K). These findings strongly indicated that FGL2 might serve as a potential biomarker for predicting the response to immune checkpoint immunotherapy.

## Discussion

Over the past few years, immunotherapy has played a critical role in the clinical treatment of BLCA, from the original development of BCG intravesical infusion to the present utilization of immune checkpoint inhibitors 33, 34. However, only a small fraction of patients can benefit from immunotherapy, and tumor progression remains a therapeutic challenge 35. Therefore, identifying accurate predictors to anticipate the immunotherapy response of patients with BLCA may assist in the rational allocation of medical resources and optimize individualized treatment strategies.

A growing body of evidence has demonstrated that FGL1 and FGL2, two members of the FREP family, play crucial roles in modulating the function of immune cells and in the development of multiple cancers 6. Nonetheless, the diagnostic value and potential function of FGL1 and FGL2 in BLCA have not been thoroughly explored. This study investigated the potential value of FGL1 and FGL2 in BLCA by analyzing their expression profiles. First, the expression levels of FGL2 were found to be significantly reduced in multiple databases and BLCA tissues, whereas no significant changes in FGL1 expression were observed. Moreover, promoter hypermethylation was one of the causes of the transcriptional silencing of FGL2 expression. Subsequently, FGL2 was determined as a protective prognostic biomarker in BLCA, as evidenced by patients with high FGL2 expression showing a better prognosis. Additionally, Cox analysis revealed that FGL2 could serve as an independent prognostic indicator for BLCA patients. Meanwhile, the nomogram model was constructed to offer a quantitative approach to predicting the prognosis of BLCA. In summary, these results highlighted that FGL2 might serve as a promising diagnostic and prognostic biomarker for BLCA.

In recent years, evidence has suggested that FGL2 functions as a novel effector molecule to exert an immunomodulatory role in a variety of tumors. For example, FGL2 accelerated glioblastoma progression by promoting the proliferation of Treg cells and the polarization of macrophages in the tumor microenvironment, exerting an immunosuppressive effect 22. Interestingly, FGL2 was also identified as a protective prognostic biomarker in breast cancer, and high FGL2 expression was positively associated with antitumor immune cell infiltration 36. Similarly, FGL2 exerted antitumor effects in lung adenocarcinoma by heightening immune cell infiltration 24. However, the function of FGL2 in the immune microenvironment of BLCA has not been confirmed until now. In this study, function enrichment analyses indicated that FGL2 was intimately associated with immune-related biological activities in BLCA. The cancer immune cycle embodies our body's immune response to cancer, it's activities in the TME collectively manifest the ultimate effects of tangle some immunoregulatory interactions 27, 37. Herein, FGL2 was found to be positively correlated with the critical steps of the cancer-immunity cycle. For instance, T-cell recruitment was significantly enhanced in the high-FGL2 group. Consistently, the infiltration levels of several TICs, such as macrophage M2, activated CD4+ memory T cells, and CD8+ T cells, were also significantly increased. Overall, these results uncovered that FGL2 might be an important regulator in tumor immunity.

Immune checkpoint blockade (ICB) is changing the treatment paradigm for many cancers by blocking the interaction between tumor cells expressing immune checkpoints and immune cells, thereby unblocking the inhibitory effect of tumor cells on immune cells 38, 39. Currently, PD1 and CTLA4 are the most studied tumor-related immune checkpoint molecules (ICPs), which have different mechanisms of action. Blocking CTLA-4 would enhance the costimulatory effect to reduce the activation threshold of T cell receptor (TCR) and may relieve the inhibitory effect of Treg cells and promote the amplification of effector T cells 40, 41. Blocking PD-1 also promotes TCR signal activation, reactivates CD8+T cells depleted by antigenic stimulation, and reprograms the tumor microenvironment to promote inflammatory rather than inhibitory myeloid cell survival 42, 43. The lack of expression of immune checkpoints results in a low response rate to ICB therapy. Emerging evidence indicates that immune checkpoint signatures can predict the prognosis and responsiveness to immunotherapy in BLCA 44, 45. The present study discovered that FGL2 expression was positively related to ICPs, indicating that BLCA patients with high FGL2 expression may have a better response to ICB treatment. High IPS was previously documented to be positively correlated with enhanced immunogenicity, predicting a better response to immunotherapy 32. The analysis of the correlation between FGL2 expression and IPS revealed that BLCA patients with high FGL2 expression exhibited higher IPS on anti-PD1 and anti-CTLA4 therapy. In addition, the results from the IMvigor210 cohort also demonstrated that patients with high FGL2 expression were more likely to benefit from immunotherapy, which further substantiated the predictive value of FGL2 on forecasting the immunotherapy effect. The above comprehensive analysis of the function of FGL2 in immune-related activities suggested that FGL2 was a promising biomarker for predicting the response to immunotherapy in BLCA patients.

Nevertheless, the limitations of the present study should be acknowledged. This analysis was conducted based on public databases and lacks further experimental validation. The specific role and underlying molecular mechanism of FGL2 in tumorigenesis and immune regulation require further research. Additionally, since protein levels may differ from RNA expression, this study mainly focused on analyzing transcriptome data and lacked validation from other datasets such as proteomics and metabolomics, which may limit the comprehensiveness of the conclusions. Besides, the predictive value of FGL2 for response to immunotherapy in BLCA could not be corroborated due to insufficient clinical data. Therefore, multi-dimensional omics sequencing should be conducted and larger-scale clinical data should be collected to further clarify the effect of FGL2 in the progression of BLCA and its predictive value in clinical treatment.

## Conclusion

Our results revealed that FGL2 was downregulated in BLCA due to promoter hypermethylation, which predicted an unfavorable prognosis. Notably, FGL2 may be a promising prognostic indicator for the immunotherapy response of patients with BLCA. Collectively, these findings help advance our understanding of the role of FGL2 and its application in the diagnosis and immunotherapy of BLCA.

## Supplementary Material

Supplementary figures.

## Figures and Tables

**Table 1:** The association between FGL2 protein levels and clinicopathological features of BLCA patients (n=71).

Characteristics	Number	Expression of FGL2		*p* value
		High (n)	Low (n)	
Gender				0.339
Male	55	17	38	
Female	16	7	9	
Age				0.083
≥60	48	13	35	
<60	23	11	12	
Tumor grade				**0.003**
Low	22	13	9	
High	49	11	38	
T stage				**0.035**
Ta+T1	32	15	17	
T2-4	39	9	30	
N stage				0.320
N0	61	22	39	
N1	10	2	8	
M stage				0.432
M0	62	22	40	
M1	9	2	7	

Numbers in bold indicate statistically significant *p* value.
