# Supplementary material for: FGL2 as a predictive biomarker for prognosis and immunotherapy in bladder cancer
Source: Int J Med Sci. 2024 May 27;21(8):1447–60. doi: 10.7150/ijms.91874 (PMC11186416; doi:10.7150/ijms.91874)
Supplement: Supplementary file 1 — Supplementary figures. [file ijmsv21p1447s1.pdf]

Supplementary file

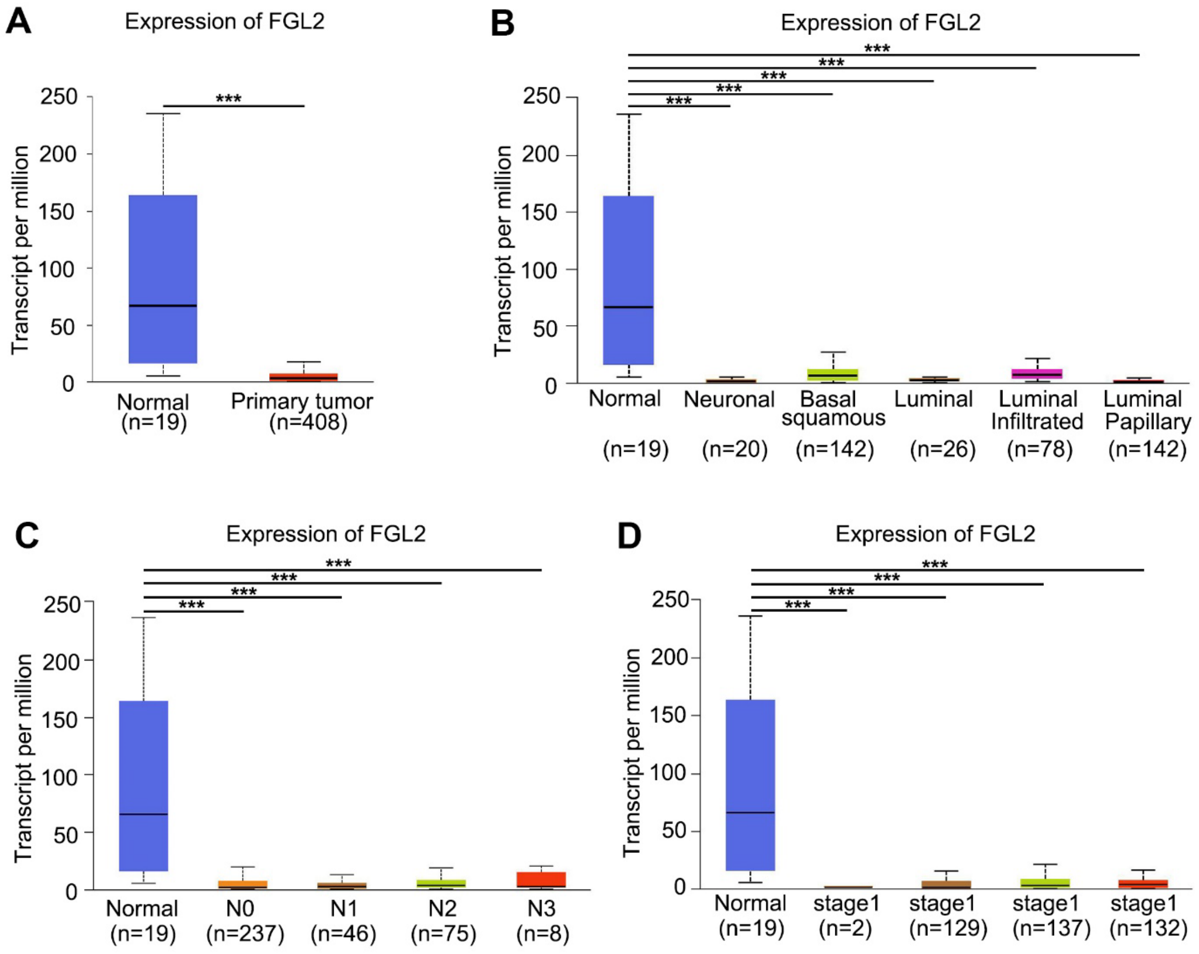

Supplementary Figure 1. The expression of FGL2 among different groups of BLCA patients based on clinical parameters. (A-D) Boxplots of FGL2 relative expression based on sample type, molecular subtype, cancer stage and lymph node metastasis in UALCAN database. ***P<0.001.

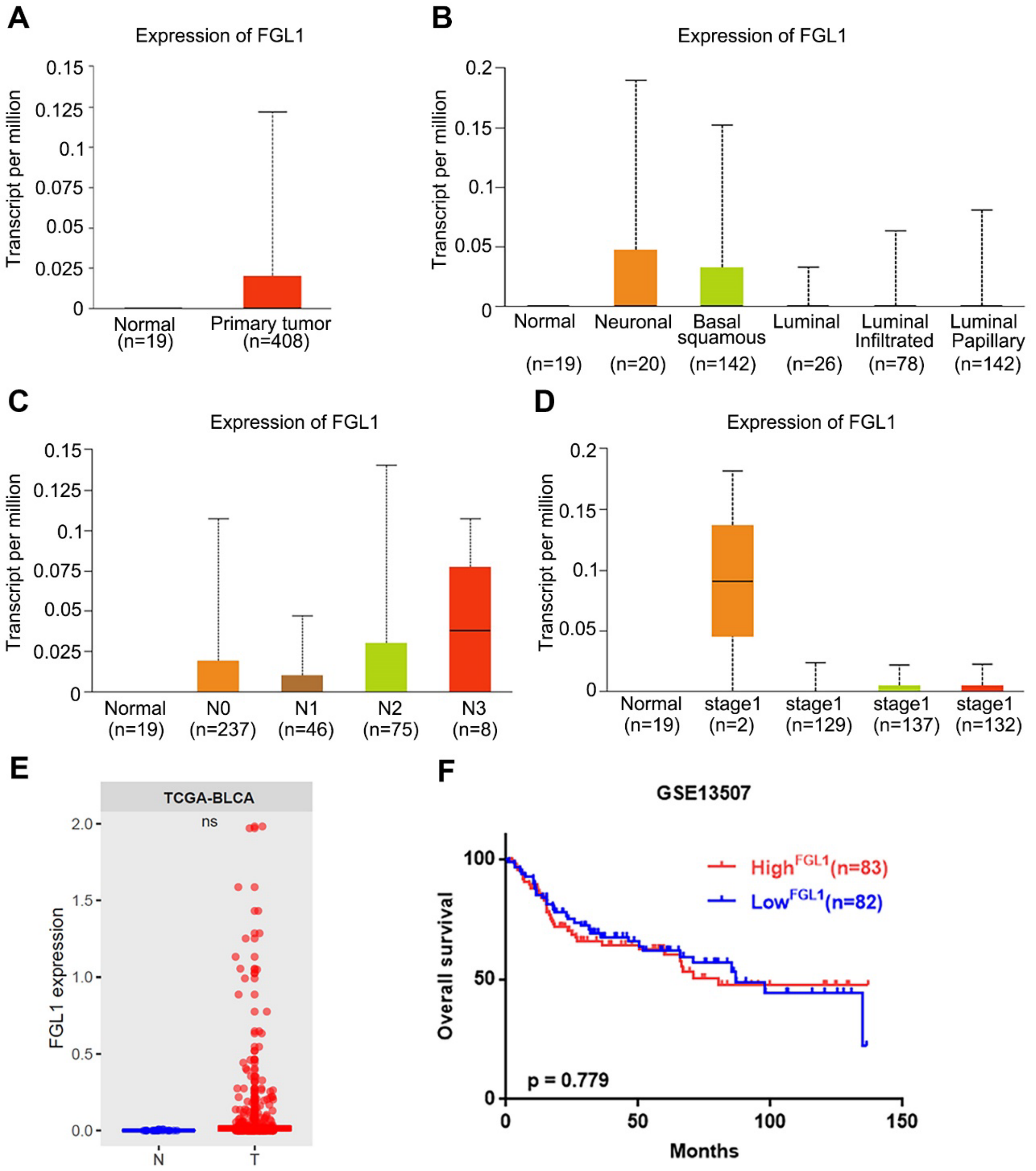

Supplementary Figure 2. The expression and prognostic value of FGL1 in BLCA patients. (A-D) Boxplots of FGL1 relative expression based on sample type, molecular subtype, cancer stage and lymph node metastasis in UALCAN database. **(E)** Boxplots of FGL1 expression difference between BLCA tissues (T) and nontumorous counterparts (N) in TCGA-BLCA. **(F)** Kaplan-Meier curve analysis of overall survival of BLCA patients in GES13507, stratified by FGL1 expression. ns. not significant.

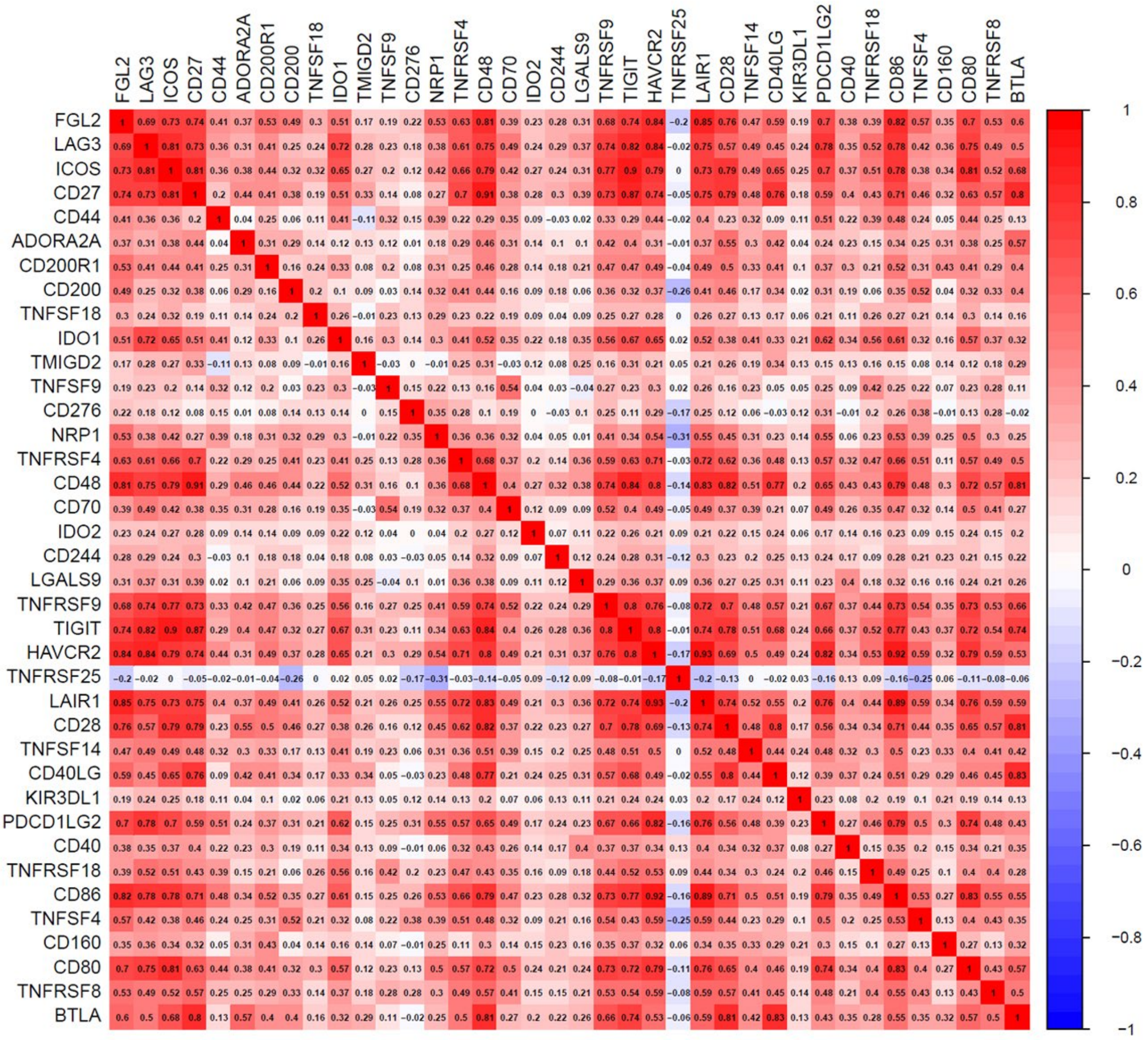

Supplementary Figure 3. The correlation of FGL2 expression of with immune checkpoint molecules (ICPs) in TCGA-BLCA.
